# The Biological Role of Conoporins, Actinoporin-like Pore-Forming Toxins from Cone Snails

**DOI:** 10.3390/toxins17060291

**Published:** 2025-06-07

**Authors:** Matija Ruparčič, Gašper Šolinc, Simon Caserman, Juan Carlos Garcia Galindo, Manuel Jimenez Tenorio, Gregor Anderluh

**Affiliations:** 1 Department of Molecular Biology and Nanobiotechnology, National Institute of Chemistry, Hajdrihova 19, 1000 Ljubljana, Slovenia; matija.ruparcic@ki.si (M.R.); gasper.solinc@ki.si (G.Š.); simon.caserman@ki.si (S.C.); 2 Doctoral Program Biosciences, Biotechnical Faculty, University of Ljubljana, Jamnikarjeva ulica 101, 1000 Ljubljana, Slovenia; 3 Departamento de Química Orgánica-INBIO, Facultad de Ciencias, Universidad de Cádiz, 11510 Puerto Real, Spain; juancarlos.galindo@uca.es; 4 Departamento de CMIM y Química Inorgánica-INBIO, Facultad de Ciencias, Universidad de Cádiz, 11510 Puerto Real, Spain; manuel.tenorio@uca.es; 5 Institut de Systématique, Évolution, Biodiversité (ISYEB), Muséum National d’Histoire Naturelle, CNRS, Sorbonne Université, EPHE, Université des Antilles, 57 rue Cuvier, CP26, F-75005 Paris, France

**Keywords:** cone snails, pore-forming toxins, actinoporin-like proteins, conoporins, conotoxins

## Abstract

Cone snails are a large group of marine gastropods that produce a complex mixture of toxic compounds to hunt prey and defend against predators. The majority of the venom comprises small toxic peptides named conotoxins, which target membrane receptors. In contrast, a smaller part of the venom contains larger proteins and conoproteins, which are thought to be involved in conotoxin maturation and the envenomation process, respectively. Interestingly, many species of cone snails contain conoporins, which are similar to actinoporins—pore-forming toxins found in sea anemones. These actinoporin-like proteins (ALPs) have recently been detected in many molluscan species, and only a few have been experimentally characterized. Due to being highly expressed in the venom gland of many cone snail species, conoporins are thought to play an important part in the envenomation process. Despite this, the exact function of conoporins is currently unknown. We propose several hypotheses aiming to elucidate their biological role.

## 1. Introduction

Cone snails are venomous marine gastropods that inhabit tropical, subtropical, and temperate waters worldwide [1]. They represent one of the most abundant lineages of marine invertebrates, with over 900 documented species that are classified into seven genera and 57 subgenera or clades according to WoRMS [2,3], or into more than 100 genera according to the classification of Tucker & Tenorio (2013) [1], which we will follow in the present work. All cone snails are carnivorous, preying on annelid worms (vermivorous), fish (piscivorous), or other molluscs (molluscivorous) [4]. They accomplish this by utilizing their venom—a complex mixture of bioactive compounds, which is injected into the prey through a modified radular tooth that is analogous to a hypodermic needle. Some piscivorous cone snails have also been observed to sedate their prey by injecting their venom into the water, which enters the circulatory system of the passing fish via uptake through gills [5]. On average, a cone snail’s venom comprises 100 to 200 toxins [6]. The venom composition differs both between specimens of different species as well as between specimens of the same species [7,8,9]. Initially, the number of all unique cone snail venom compounds was estimated to be around 100,000 [10,11]. However, that number has since been re-evaluated and is now believed to exceed one million [12,13,14].

The majority of the venom comprises small cysteine-containing peptides, conotoxins, which generally contain between 10 and 30 amino acids in their mature form and are connected by disulphide bonds in a highly conserved fashion, giving rise to highly conserved architectures named as frameworks. They are synthesized as inactive precursors, consisting of an N-terminal signal peptide followed by a propeptide and a C-terminal mature peptide sequence [6,15]. Following enzymatic cleavage and chemical modification, the mature peptide can exert its toxic activity by binding specifically to membrane proteins, such as ion channels, G-protein coupled receptors, or neurotransmitter transporters [16,17].

In addition to the low molecular weight conotoxins, cone snail venom also contains high molecular weight components, called conoproteins, the function of which remains poorly understood [18]. Some venom proteins are believed to be involved in the maturation of conotoxin precursors, such as proteinases [19,20,21], arginine kinases, peptidyl-prolyl *cis*-*trans* isomerases, and protein disulfide isomerases [11,22], while conoproteins are thought to be involved in the envenomation process. These conoproteins include conodipines with phospholipase-A_2_ activity [23], hyaluronidases (conohyals) [24], and conoporins, which are similar to actinoporins, pore-forming toxins from sea anemones [22,25]. In this review, we first briefly summarize the current knowledge of actinoporins. Next, we describe the characteristics of actinoporin-like proteins found in molluscs, focusing on the widespread occurrence in cone snails. Finally, we conclude this review with hypotheses regarding the biological role of conoporins in cone snail venoms.

## 2. Actinoporins

**Figure 1:**
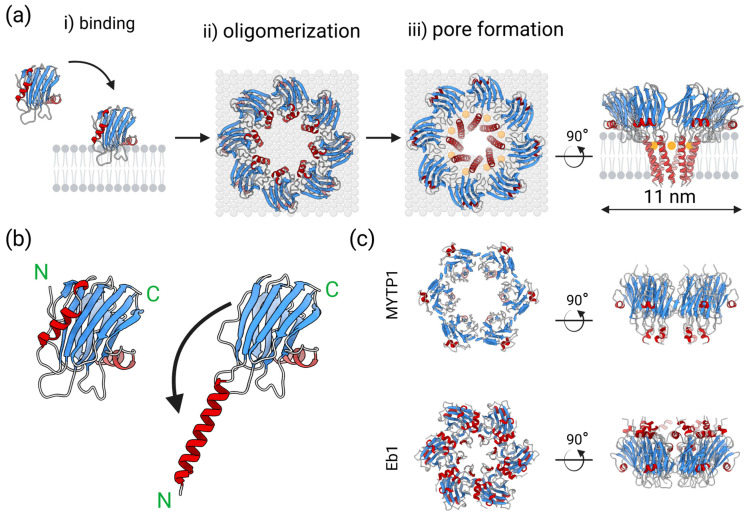
Pore formation by actinoporins. (**a**) (i) Water-soluble monomers (PDBid: 3LIM [31]) bind membranes containing sphingomyelin. (ii) Monomers oligomerize at the plane of the membrane. (iii) A total of 7–9 protomers form the final funnel-shaped pore (PDBid: 4TSY [37]). Orange circles represent lipids involved in the pore structure. (**b**) During pore-formation, the N-terminal α-helix detaches from the β-sandwich and elongates. (**c**) Top and side views of the low-resolution cryo-EM reconstruction of MYTP-1 pore [40], and Alphafold3 (AF3) [41] model of hexameric Eb1 from *Virroconus ebraeus*.

Formation of pores in lipid membranes is a widespread strategy of organisms in attack or defense [26]. Actinoporins are a family of α-pore-forming toxins from sea anemones with a molecular weight of about 20 kDa [25,27]. They are components of the venom that sea anemones use to paralyze and digest prey, as well as to defend against predators. The toxic effects of actinoporins are exerted by the formation of transmembrane pores in target cells [28], which proceeds by a succession of well-defined steps: binding to the lipid membrane, oligomerization at the plane of the membrane, and, finally, formation of transmembrane pores (Figure 1a). Actinoporins from different anemone species share a highly conserved amino acid sequence as well as a three-dimensional structure. All known structures of actinoporins (equinatoxin II (EqtII) from sea anemone *Actinia equina* [29,30], fragaceatoxin C (FraC) [31] and fragaceatoxin E (FraE) [32] from *Actinia fragacea*, sticholysin I (StnI) [33] and sticholysin II (StnII) [34] from *Stichodactyla helianthus*) share a conserved fold with very few differences [25]. Actinoporins monomers consist of a compact β-sandwich of 10 β-strands surrounded by two α-helices. The N-terminal part of approximately 30 amino acid residues, which also includes the N-terminal α-helix, is a region that can adopt alternative conformations without breaking the β-sandwich fold (Figure 1b) [34]. A hallmark of actinoporins is their high affinity for membrane lipid sphingomyelin [25,35], which serves as a high-affinity receptor, with individual monomers binding at least four sphingomyelin molecules to lipid binding sites L2–L5 [36]. Sphingomyelin at position L1 is crucial for triggering pore formation and is also a structural lipid and an integral part of the pore structure [37]. In addition to sphingomyelin, actinoporins can bind to ceramide phosphoethanolamine [38] and phosphatidylcholine [39], although their pore-forming activity significantly depends on the presence of sphingomyelin in target lipid membranes [28]. The lipid-binding region of monomers under the C-terminal helix is well suited for binding up to six different lipid molecules simultaneously [36,37]. After membrane binding, monomers oligomerize and form a transmembrane channel with a cluster of amphipathic N-terminal helices from seven to nine protomers (Figure 1a).

**Figure 2:**
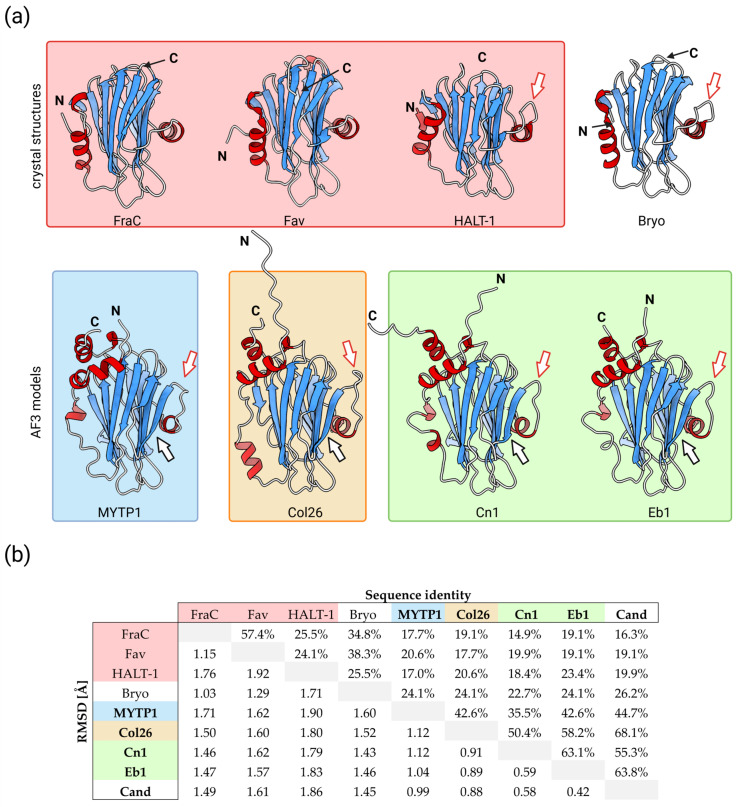
(**a**) Side-by-side comparison of actinoporin FraC and some examples of actinoporin-like proteins. Crystal structures of FraC (PDBid: 3LIM), dN53Fav (PDBid: 9EYL), HALT-1 (PDBid: 7EKZ), and Bryoporin (PDBid: 7PUD) are shown in the top row. The bottom row shows AF3 [41] prediction models of ALPs from molluscs. From left to right: MYTP1 (*Mytilus galloprovincialis*), Col26 (*Cumia reticulata*), Cn1 (*Pionoconus consors*), and Eb1 (*Virroconus ebraeus*). N- and C-termini are marked, and cartoons are colored according to the secondary structure. Red arrows highlight the extension of a loop present in most ALPs, and the black arrow marks the additional strand present in ALPs from molluscs. Areas are shaded according to the organism of origin: red, cnidarian actinoporins and ALP; blue, mytiporins; orange, coluporins; green, conoporins. (**b**) Root mean square deviation (RMSD) and sequence identity comparison of the actinoporins and ALPs from panel (**a**). The values were calculated using PDBeFold v2.59 [55]. The proteins on which the AF3 model was used for the calculations are shown in bold text. Additionally, the AF3 model of Cand is shown to represent the only currently characterized conoporin.

Proteins with a similar structure, but not necessarily a similar amino acid sequence, to that of actinoporins are termed actinoporin-like proteins (ALPs) (Figure 2) [42,43,44,45]. ALPs are common in other cnidarians, such as HALT (hydra actinoporin-like toxins) from *Hydra magnipapillata* [46,47], which share 30–36% sequence identity with actinoporins [48]. The best characterized is HALT-1, which differs from typical actinoporins in its structure, containing an extension in the loop preceding the second α-helix (Figure 2) and forming pores with a larger diameter (20–30 Å compared to 11–12 Å of FraC), as well as in its function, exhibiting a lower hemolytic activity compared to actinoporins [49,50]. ALPs are also found in oomycetes [44], fungi [45,51], fish [42], plants [38,42], and even in bacteria [52,53,54].

## 3. Actinoporin-like Proteins in Molluscs

The first ALPs from molluscs were isolated from the salivary gland of the predatory hairy triton *Cymatium* (*Monoplex*) *parthenopeum* form *echo* (family Ranellidae), and were named echotoxins [56]. These 25 kDa proteins were shown to exhibit strong hemolytic and lethal activity in mice, which was inhibited by the addition of gangliosides. In contrast, an almost 300-fold amount of sphingomyelin was required to achieve the same effect, leading to the conclusion that the receptor for echotoxins is most likely gangliosides and not sphingomyelin, as in typical actinoporins [56,57]. Following the rise of next-generation sequencing technologies, additional ALPs were discovered in the transcriptomes and proteomes of other molluscs, such as in the giant triton *Charonia tritonis* [58], the drilliids *Clavus canalicularis* and *Clavus davidgilmouri* [59], and the terebrids *Terebra anilis* and *Terebra subulata* [60]. In the brackishwater clam *Corbicula japonica*, three ALPs were isolated from the foot muscle, called clamlysins. Out of them, clamlysin B was shown to bind specifically to sphingomyelin, making it a potential sphingomyelin-detecting probe [61]. Recently, five ALPs were discovered in the transcriptomes of marine gastropods of the genus *Littorina*. These so-called littoporins (LitP) were predicted to have a similar secondary structure to other ALPs, with LitP-4 containing an unusually long N-terminal extension, containing an interferon-induced 6–16 family domain IFI6/IFI27 flanked by two disordered regions [62].

**Figure 3:**
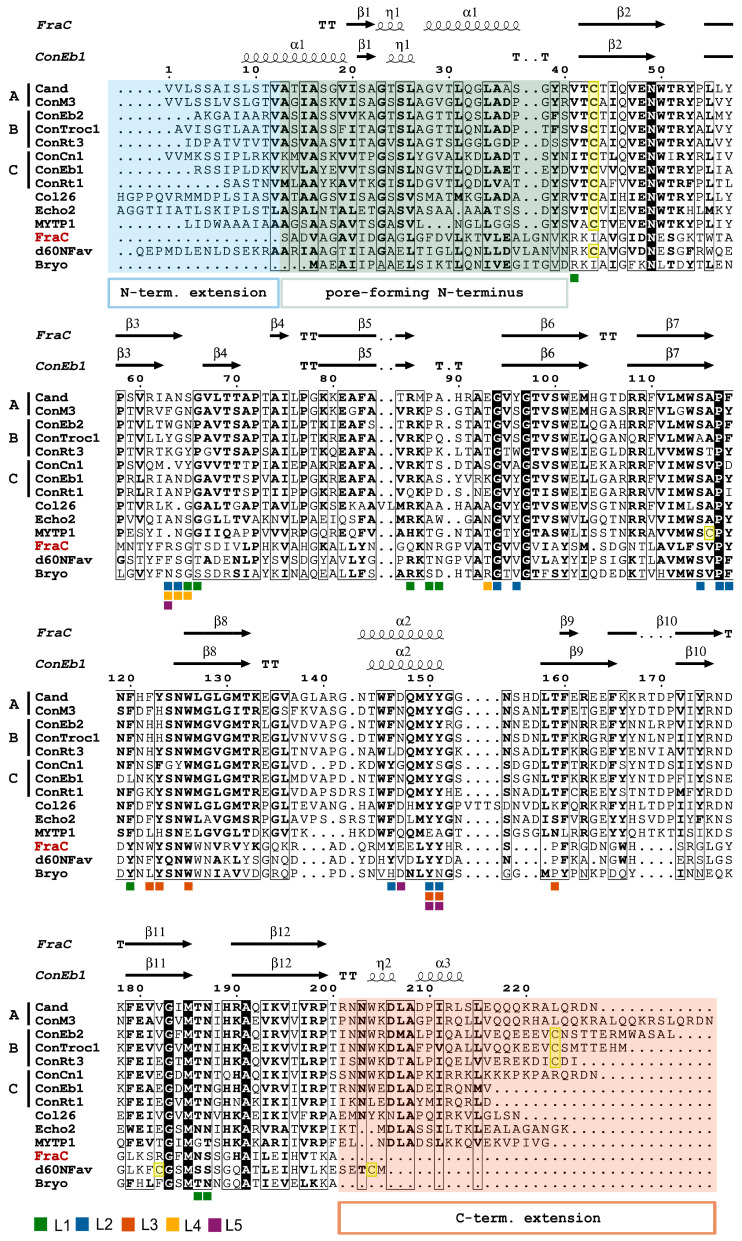
Multiple sequence alignment of actinoporin FraC and thirteen selected actinoporin-like proteins. Bryo, bryoporin; Cand, conoporin from *Turriconus andremenezi*; Col26, coluporin 26; Con, conoporin; Cn, *Pionoconus consors*; Eb, *Virroconus ebraeus*; M, *Pionoconus magus*; Rt, *Rhizoconus rattus*; Troc, *Kalloconus trochulus*; Echo2, echotoxin-2; MYTP1, mytiporin-1; Fav, actinoporin-like protein from *Orbicella faveolata*. The secondary structures of FraC (PDBid: 3LIM) and Eb1 (AF3 [41] model) are shown. Fav, which contains a very long N-terminal extension, is shown without the first 60 residues. The N- and C-termini extensions, the region of the molecule that participates in pore formation, and cysteines are highlighted in blue, pink, green, and yellow, respectively. Conoporins are additionally labelled by their phylogenetic clade (Figure 4). Colored squares denote amino acid residues from Fav that are crucial for binding to the target membrane via interaction with lipids L1–L5. The residues that interact with lipid L6 are not shown, as they are not crucial for membrane binding [36].

Similar to cnidarian ALPs, molluscan ALPs do not share high sequence similarity with actinoporins, showing poor conservation of amino acid residues that are crucial for sphingomyelin binding in actinoporins (Figure 3). At the same time, their secondary structure is predicted to be highly similar to an actinoporin fold, consisting of a β-sandwich flanked by two α-helices. The main characteristics that differentiate the structure of molluscan ALPs from typical actinoporins are as follows: (i) Extension at the N-terminus; (ii) The presence of extra β-strands in the β-sandwich; (iii) Extension in the loop preceding the second α-helix; (iv) Extension at the C-terminus (Figure 2); (v) The presence of cysteines, which are not found in actinoporins [40,56,62,63].

**Figure 4:**
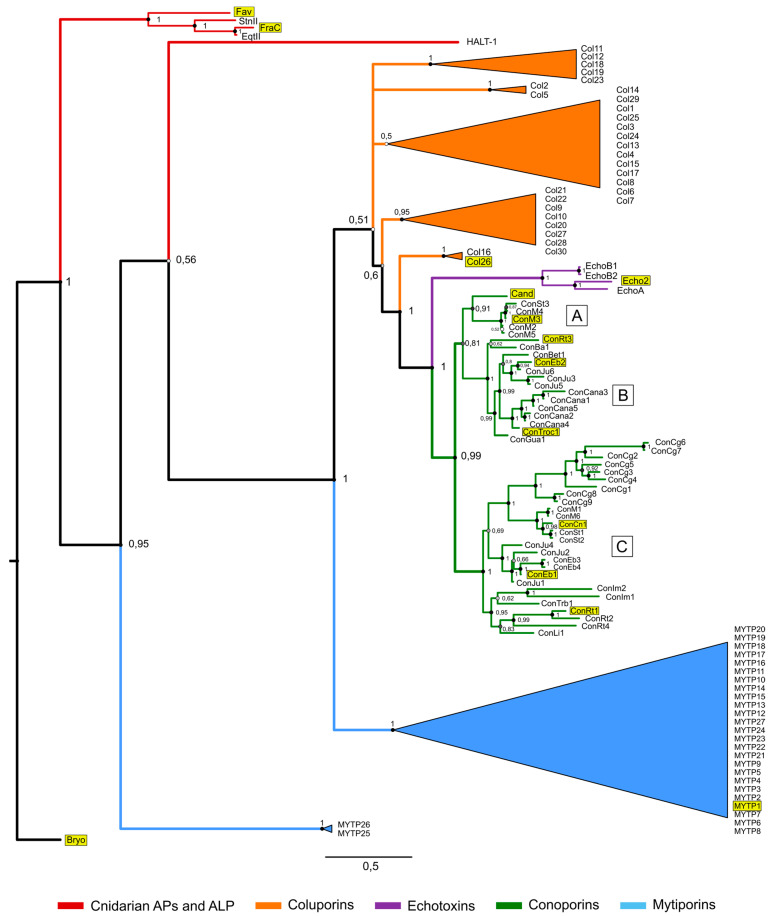
Bayesian phylogeny of actinoporins and ALPs. Nodes are colored according to the posterior probability value. Clades containing sequences from the same species are collapsed. The sequences used in the alignment (Figure 3) are highlighted in yellow. Bryoporin from the moss *Physcomitrella patens* was used as an outgroup. The three clades formed by conoporin sequences are marked by letters in squares. FraC, fragaceatoxin C; EqtII, equinatoxin II; StnII, sticholysin II; HALT-1, hydra actinoporin-like toxin 1; Col, coluporin; Cand, *T. andremenezi*, Con, conoporin; Ba, *Stellaconus bayani*; Bet, *Dendroconus betulinus*; Cana, *Kalloconus canariensis*; Cg, *Gastridium geographus*; Cn, *Pionoconus consors*; Eb, *Virroconus ebraeus*; Gua, *Varioconus guanche*; Im, *Rhombiconus imperialis*; Ju, *Virroconus judaeus*; Li, *Lividoconus lividus*; M, *Pionoconus magus*; Rt, *Rhizoconus rattus*; St, *Pionoconus striatus*; Trb, *Kioconus tribblei*; Troc, *Kalloconus trochulus*. The full-length sequences were aligned with Clustal Omega v1.2.4 [64], and the alignment was used to construct a phylogenetic tree in MrBayes v.3.2.4 [65] using an MCMC analysis with the WAG + G model, which was predicted to be the most suitable model by the software Mega 11 [66] and 2,000,000 generations.

The two mollusc species with the highest number of ALP sequences discovered to date are the Mediterranean mussel *Mytilus galloprovincialis* with 27 mytiporins and the colubrarid vampire snail *Cumia intertexta*, with 30 coluporins, showing a remarkable expansion of ALP genes in these species (Figure 4) [40,63]. In *Mytilus*, mytiporin-1 (MYTP1) was biochemically characterized and was shown to exhibit weak hemolytic activity on bovine erythrocytes. In contrast to typical actinoporins, which form octameric pores on sphingomyelin-containing membranes, the pores formed by MYTP1 were hexamers (Figure 1c), forming only on membranes containing 1-palmitoyl-2-oleoyl-*sn*-glycero-3-phosphoglycerol (POPG) in the absence of sphingomyelin, which is the consequence of MYTP1 not having conserved residues that are crucial for sphingomyelin-binding in typical actinoporins [40]. As for *Cumia*, many coluporins were found to be significantly expressed in the salivary gland, leading to the assumption that they aid in host tissue penetration during hematophagy [63]. Of these, coluporin-16 and coluporin-26 were shown to exhibit very weak hemolytic effects on bovine erythrocytes. Similar to MYTP1, they bound most efficiently to POPG-containing vesicles, with coluporin-26 forming hexameric pores [40].

## 4. Conoporins

Ten years after the discovery of echotoxins, Terrat et al. discovered an ALP-encoding sequence in the venom gland transcriptome of the piscivorous cone snail *Pionoconus consors* [11]. In the same year, Violette et al. confirmed the presence of the ALP both in the *P. consors* transcriptome as well as in the proteome and named it conoporin-Cn1. The 25 kDa protein was shown to be 36% identical to echotoxin-2 and was detected in nine different isoforms, which corresponded to different oxidation states [67]. Both studies showed conoporin-Cn1 to constitute a significant part of the *P. consors* venom, comprising approximately 4% of the venom gland transcriptome and 9% of the venom proteome [11,67].

With the rise of NGS analyses of venom composition, several other cone snails were found to contain conoporins in their venom. We have built up a database containing all the transcriptomes of cone snails published [68] and conducted a systematic search for conoporins. To date, 95 unique conoporin sequences have been detected in 27 cone snail species (Figure 4), which belong to 13 different clades (Table 1). More than a quarter of all detected conoporin sequences (26.3%) were found in cone snails from the *Virroconus* clade, while the *Lautoconus* clade contains the highest number of species encoding conoporins (5). The majority of conoporin sequences were detected in cone snails with a vermivorous diet (71.6%), while the rest were detected in piscivores (28.4%). To date, no conoporin sequences have been detected in cone snails with a molluscivorous diet. This might be connected to the different hunting strategy used by molluscivorous cone snails, which have been observed to sting their prey several times before fully incapacitating it [69], a possible adaptation for hunting slow-moving prey that does not require rapid immobilization [70]. Interestingly, two conoporin transcripts were detected in the venom duct transcriptome of the vermivorous *Rhombiconus imperialis* [71], which has a specialized diet on venomous fireworms and was shown to have a venom profile more similar to those of molluscivorous cone snails [72]. Surprisingly, while many molluscs have been found to express PFTs from families other than actinoporins [73,74,75,76,77], none of them have so far been detected in cone snail transcriptomic and proteomic studies, suggesting that conoporins are the sole PFT in cone snails.

When analysed on a phylogenetic tree, the full-length sequences of conoporins appear to form at least three distinct clades: A, B, and C (Figure 4). The main driver separating conoporin sequences into different clades are the N- and C-termini, where the most sequence variety is observed. At the same time, the core β-sandwich seems relatively well conserved, which is also apparent in the alignment shown in Figure 3. A deeper look at the tree shows that some species of cone snails have conoporins on more than one clade. This is observed in the piscivorous *P. magus* and *P. striatus* (clades A and C), and in the vermivorous *R. rattus*, *V. ebraeus*, and *V. judaeus* (clades B and C), suggesting that there might be two types of conoporins in each species. It is also worth noting that in clade C, all conoporins from the piscivorous *G. geographus*, *P. magus*, *P. striatus*, and *P. consors* occupy their own sub-clade, while the ones from vermivorous snails are grouped in the other two sub-clades.

The majority of conoporins were discovered in the venom gland transcriptomes, where some were found to be moderately expressed (1–40 transcripts per million (TPM) in *Virroconus coronatus* [71]), while others showed extremely high relative expression values (e.g., 6457 and 9172 TPM for conoporins from *R. imperialis* [72]). Additionally, some conoporins have also been confirmed at the protein level. These include conoporins from the two sister species *V. ebraeus* and *V. judaeus*, which constitute 0.6% and 0.8% of the venom duct proteome, respectively [68]. In *G. geographus*, nine conoporins, termed conoporin-Cg1–9, were detected. Despite originating from the same species, some were shown to vary both in the primary sequence as well as in the isoelectric point, which ranged from 6.3 to 9.6. Four of them (conoporin-Cg1–4) were confirmed both at the transcript and protein level, while the remaining five (conoporin-Cg5–9) could not be resolved on pH 4–7 strips used in 2D-electrophoresis due to their basic pI values [90]. Interestingly, in the comparative analysis of the venom transcriptome and proteome of the juvenile and adult *P. magus*, no conoporins were detected in the juvenile snails, however, conoporin-M6 was detected in both the transcriptome, constituting 13% of the distal venom gland transcripts, as well as in the proteome of the adult snails [88].

For now, the only conoporin to be experimentally characterized at the protein level is Cand from the vermivorous *T. andremenezi*, where it was shown to represent 0.02% of the total venom gland transcriptome [85]. The mature form consists of 227 amino acid residues and shares 23% identity with FraC, with the majority of residues crucial for sphingomyelin-binding conserved. Additionally, it contains an 11-amino acid extension at the N-terminus and as well as a 27-amino acid long C-terminal extension, which is predicted to form an α-helix (Figure 3). While Cand was shown to bind strongly to multilamellar vesicles composed of a 1:1 ratio of 1-palmitoyl-2-oleoyl-*sn*-glycero-3-phosphocholine (POPC):SM, similar to FraC, it differed from typical actinoporins by strongly binding to membranes composed of POPC:cholesterol (1:1) and POPC as well, exhibiting non-specific binding to target membranes. Similar to other characterized molluscan ALPs, Cand formed hexameric pores on large unilamellar vesicles composed of POPC:SM (1:1) [40].

It is currently unknown whether conoporins exert toxicity alone or in synergy with other toxins. With the discovery of the first conoporin-Cn1 from the piscivorous *Pionoconus consors*, Violette et al. [67] hypothesized that conoporins could participate with conohyals in disrupting the cellular membrane and extracellular matrix, respectively, thereby permeabilizing prey tissue and accelerating the envenomation process. Recently, Koch et al. [72] conducted an exhaustive bioinformatic analysis of 42 different cone snail venom gland transcriptomes, from which they obtained information on potential gene co-expression through hierarchical clustering of conotoxin gene superfamily expression values. The hierarchical clustering yielded three groups, with conoporins falling into group 2 together with 27 other conotoxin superfamilies, many of which have an unknown function. Within this group, conoporins were coupled closely with the superfamilies S, MKIYL, conopressins, conodipines, and conohyals. Moreover, principal component analysis (PCA) using superfamily expression data showed that conoporins, together with conohyals, conodipines, conopressins, and MKYIL, are a major driver of piscivore venom separation [72].

## 5. Biological Role of Conoporins

Despite conoporins being abundantly present in the venom of several cone snail species, their exact function remains unclear, owing largely to the lack of experimentally characterized conoporins thus far. Nevertheless, we propose several hypotheses about their biological role to elucidate the exact function of these pore-forming toxins (Table 2). These hypotheses are based on the current knowledge of other pore-forming toxins that were discovered to have similar roles to the ones proposed for conoporins.

### 5.1. Disruption of Cells and Epithelia

**Figure 5:**
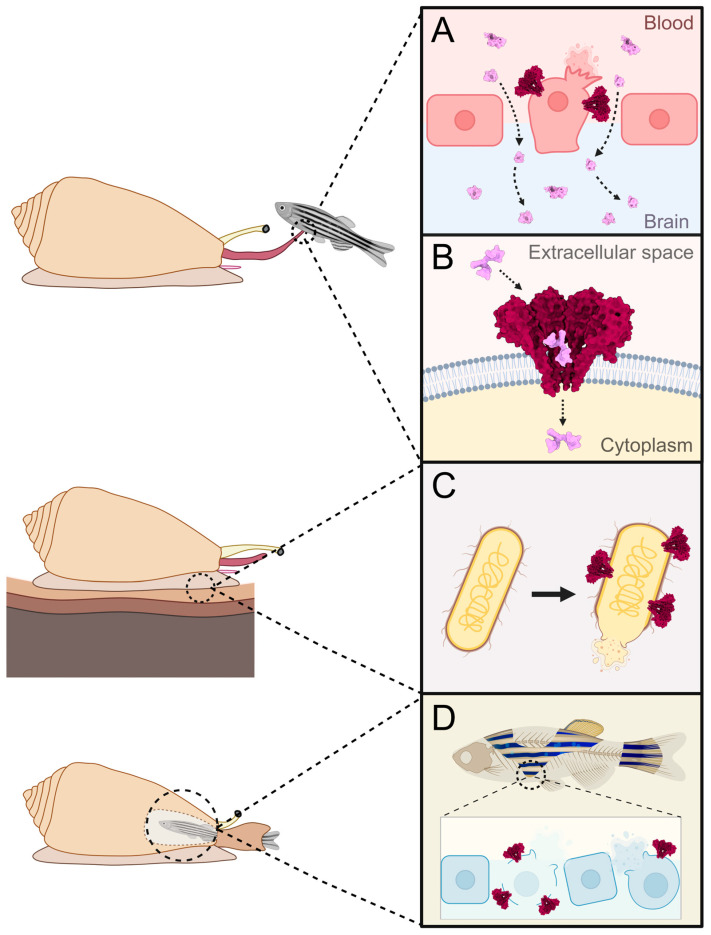
Possible biological roles of conoporins. Each box illustrates one of the hypotheses regarding the function of conoporins. Once the venom is injected into the prey (e.g., fish), conoporins can aid in the envenomation in the following ways: (**A**) Destroying tissue by causing cell lysis via pore formation, which can lead to permeabilization of endothelial barriers (e.g., blood–brain barrier), allowing smaller conotoxins to pass through the barrier; (**B**) Acting as gates, allowing for the translocation of conotoxins into the cell through the formed pore; (**C**) Conoporins that are secreted in the mucus of the foot could have an anti-microbial role, destroying pathogens through pore-induced lysis. (**D**) If conoporins are also expressed in the digestive system, they could facilitate digestion by lysing cells of the ingested tissues. Created with Biorender.com.

The first hypothesis suggests that pore formation by conoporins, much like that with actinoporins and other PFTs [120,121,122] (Figure 5A), causes the targeted cell to swell due to colloid-osmotic shock, leading to cell lysis. For cone snails, this could be beneficial for the envenomation process as cell lysis and tissue degradation caused by conoporins would accelerate the spread of the smaller conotoxins through the body of the prey. The findings of Koch et al. [72] further support this hypothesis, since conoporins from piscivores seem to co-express with conohyals and conodipines, while several vermivorous conoporin-expressing snails were found to express both conoporins and conodipines, indicating a potential role of these proteins in the destruction of cell membranes and tissue. Furthermore, if conoporins were to form pores on epithelial cells, the resulting cell swelling and lysis could loosen tight junctions between cells, enabling venom components to pass through the barrier. This mode of action has already been described for many PFTs. For the α-helical PFT EqtII and the β-barrel PFT listeriolysin O (LLO), transepithelial electrical resistance (TEER) measurements showed that both toxins cause paracellular permeabilization of Caco-2 epithelial monolayers by causing a rearrangement of tight junction proteins [92]. While EqtII and LLO were observed to permeabilize Caco-2 epithelia for ions only, two β-barrel PFTs, anthrolysin O (ALO) and aerolysin, were shown to increase paracellular permeability for larger molecules as well, with ALO permeabilizing Caco-2 monolayers to 3 kDa FITC-dextran [93], and aerolysin allowing for paracellular transport of 4 kDa FITC-dextran through HT-29/B6 monolayers [94]. Since conotoxins target membrane proteins on nerve cells, the barrier that would be especially important for them to cross is the blood-brain barrier, where conoporins could facilitate this process by permeabilizing this endothelium (Figure 5A). PFTs have been reported before to have the ability to compromise the blood-brain barrier. The most researched PFT with this effect is the β-barrel PFT epsilon toxin (ETX) from *Clostridium perfringens*, which was shown to disturb the integrity of the blood–brain barrier, allowing for the passage of not only itself, but also of other molecules [95,96,97,99]. Moreover, on a zebrafish humanized model of the blood-brain barrier, Adler et al. [98] recently demonstrated that high concentrations of ETX can cause barrier disruption to the extent of allowing the passage of extremely large macromolecules, such as 2000 kDa dextrans [98]. Since most conotoxins are short peptides with low molecular mass, a conoporin-induced blood–brain barrier permeabilization much lower than that of ETX would already suffice for the passage of conotoxins through the barrier towards the central nervous system (Figure 5A). All of this considered, it is a possibility that during the envenomation of vertebrates, conoporins could act in the permeabilization of crucial endothelial barriers. Interestingly, a study by Jackson et al. [104] showed that the actinoporin EqtII, when present at sublytic concentrations, forms pores on *Plasmodium falciparum*-infected human red blood cells that have a diameter up to 100 nm, which is substantially larger than a typical octameric EqtII pore, which has a diameter of 1–2 nm [123]. These larger secondary pores, therefore, allow for the entry of proteins into the cell and were used by Jackson et al. to translocate antibodies into erythrocytes for immune labelling [104]. If conoporins were able to induce similar secondary pores with a large diameter, these could allow for the transcellular passage of even larger venom components, such as conoproteins.

### 5.2. Entry of Small Conotoxins into Cells Through Pores

The second hypothesis suggests that conoporins act as gates to cells, enabling rapid passage of smaller conotoxin peptides into the cell through the formed pore (Figure 5B). The process of PFT-aided protein translocation has previously been described in several cases. For example, in *Bacillus anthracis*, where the anthrax toxin protective antigen (PA) was shown to form pores that enable the passage of the edema factor (EF) and lethal factor (LF) proteins into the cytosol [100,101]. *Streptococcus pyogenes* utilizes streptolysin O β-barrel PFT to translocate the SPN (*S. pyogenes* NAD-glycohydrolase) effector protein into the target cell cytosol [102,103]. And finally, in the human perforin/granzyme pathway, where pores formed by perforin allow for the entry of the granzyme serine protease into the target cell, inducing apoptosis [105,106]. Interestingly, some conoporin-expressing species also express conotoxin gene superfamilies that contain short peptides with unknown function [72]. If some of these were to have an intracellular target, conoporins could act as the doors that allow for their entry into the target cell.

It has been suggested that in vermivorous cone snails, some venom components serve as a defense against fish predation and may represent a pathway for the evolutionary transition from the vermivorous to the piscivorous feeding strategy [124]. This assumption is supported by the discovery that the δ-conotoxin SuVIA of the vermivorous *Tesselliconus suturatus* activates voltage-gated sodium channels in vertebrates and triggers pain sensations [125]. The toxin is expressed in the proximal part of the snail’s venom gland, suggesting a defensive function. The perception of pain in vertebrates involves terminals of neurons known as “nociceptors” [126]. They recognize and react quickly to mechanical, thermal, or chemical stimuli of sufficient intensity to cause tissue damage or whose quality indicates existing tissue damage. When activated, the neurons transmit this information to the spinal cord and brain via action potentials. Actinoporins are known to damage cells by making cell membranes permeable to cations. This can lead to increased excitability and contraction of myocytes [127,128], indicating a high potential to activate pain perception. Actinoporins are also thought to play a role in the predation and defense of sea anemones. Therefore, we hypothesize that conoporins, similar to δ-conototoxins, may also function by inducing a transient pain sensation that is likely reflected in strong twitching of the fish in the first few seconds after injection of the cone snail venom. We hypothesize that these strong contractions promote the distribution of the venom in the body and accelerate the systemic effects of all venom components. Interestingly, in *Pionoconus magus*, which feeds exclusively on polychaetes as juveniles while eating fish as adults, conoporin was shown to be expressed only in the adult stage [88]. In addition, conoporins were found to be a very common venom component in piscivorous cone snails, but are absent in molluscivorous cone snails [72], further emphasizing the importance of conoporins in the hunting of fish by cone snails. Furthermore, it is suggested that cephalopods, regarded as molluscs with the most developed central nervous system, do not feel pain [129], which would render conoporins redundant for mollusc hunting if they were to function in pain induction. Finally, pain sensations are also known from records of injuries to humans by cone snails [130]. Intense localized initial pain is typical of the stings of many piscivorous cone snails, but is rare in molluscivorous snails.

### 5.3. Part of the Immune System

In addition to envenomation, conoporins could also be involved in other physiological processes, such as innate immunity (Figure 5C). One of the most notable instances of an antimicrobial PFT is lysenin, an aerolysin-like β-barrel PFT from the earthworm *Eisenia fetida* [107,108]. The toxin is secreted into the coelomic fluid, where it acts to defend against parasitic microorganisms by forming nonameric pores on sphingomyelin-containing target membranes [109,131]. PFTs with antimicrobial activity were also described in molluscs. Many of them show similarity to the membrane attack complex and perforin (MACPF) family [110,111], which is an important part of the innate immune system of vertebrates. Similar to their vertebrate counterparts, MACPFs from molluscs have also been shown to act in the immune system, protecting them from bacteria [75,112,113] and parasites [76]. A particularly noteworthy case of the use of MACPFs was discovered in the gastropod *Pomacea canaliculate*, where two proteins, one similar to MACPFs and the other to tachylectins, form a heterodimer, called PmPV2. The two subunits are expressed in the eggs, suggesting a defensive role, whereby upon ingestion of the eggs by a predator, the PmPV2 toxin disrupts the digestive and nervous systems of the predator [114]. In addition to MACPFs, proteins from other PFT families have also been shown to be involved in the immune system of molluscs. In the genus *Mytilus*, two proteins belonging to the recently described mytilectin family [115], named MytiLec-2 and -3, were discovered to contain an aerolysin-like pore-forming domain at the C-terminus of the protein. It is hypothesized that MytiLec-2 and -3 act in an anti-bacterial response with MytiLec-1, which does not contain a pore-forming domain, whereby MytiLec-1 recognizes and opsonizes the pathogen, while MytiLec-2 and -3 destroy it by forming pores with their aerolysin-like domain [116]. In the freshwater snail *Biomphalaria glabrata*, a total of 21 aerolysin-like proteins, termed biomphalysins, were discovered, many of which were found to be upregulated when the snail was exposed to bacteria, yeast, and parasites [117]. Of these, biomphalysin 1 was characterized in detail, where it was found to bind to the membrane of *Schistosoma mansoni* sporocysts, causing lysis of the parasite cells [73]. It was later discovered that *B. glabrata* also encodes five proteins with a fold similar to β-barrel PFT Cry toxins from *Bacillus thuringiensis*, which were named glabralysins. Similar to biomphalysins, most glabralysins also showed an increase in gene expression upon infection with bacteria, yeast, and parasites [74], further solidifying the importance of PFTs in the immune system of this gastropod. In addition to β-barrel PFTs, ALPs have also been shown to have a potential role in the immune response, namely in gastropods of the genus *Littorina*. In *Littorina littorea*, it was discovered that two littoporins, LitP-1 and LitP-2, were upregulated in hemocytes and kidney tissue upon infection with the trematode *Himasthla elongata*, respectively, indicating a potential role in the anti-trematode immune response [62]. Since the current transcriptomic and proteomic data of cone snails report no expression of any other PFTs, it is possible that some conoporin isoforms could exert an anti-pathogen effect. The best candidate for this is the vermivorous *L. ventricosus*, where transcriptomic data showed a conoporin having a two-orders-of-magnitude higher expression rate in the foot than in the venom gland [80], implying a possible antimicrobial role (Figure 5C).

### 5.4. Digestion

Lastly, conoporins could also have a role in digestion (Figure 5D), as has been described with pore-forming toxins from other organisms. The best studied examples are hydralysins, aerolysin-like β-barrel PFT from the green hydra, which have been shown to be produced in endodermal digestive cells and secreted into the gastrovascular cavity upon prey engulfment [118]. The toxin binds to the cell membranes of the ingested prey, but not to the hydra membranes, forming pores that cause osmotic disintegration of prey tissue [119]. A digestive role has also been proposed for the actinoporin-like mytiporins, which were found to be highly expressed in the digestive gland, suggesting their involvement in digestion [40]. However, since most cone snail transcriptomics is performed using tissue from the venom apparatus, the digestive role hypothesis cannot be evaluated until transcriptomic data from digestive and other tissues are available.

It has been shown that many cone snail species encode more than one type of conoporin. This could be part of a hunting strategy, where each conoporin isoform evolved to bind to a different target membrane, expanding the repertoire of prey species the snail can successfully immobilize with its venom [63,90]. Since the venom of some conoporin-absent cone snails was shown to vary in its contents depending on whether the venom is used in an attacking or defending manner, the same could also be true for conoporin-expressing species, where different conoporins could be used either to hunt prey or defend against predators [132]. Lastly, different isoforms of conoporins could have different functions altogether (e.g., envenomation, immune system, digestion).

## 6. Conclusions

In toxinology, cone snails represent an extraordinary group of animals, producing a wide repertoire of bioactive toxic compounds. While the majority of cone snail toxin studies tend to focus on the low molecular weight conotoxins, research on the high molecular weight conoproteins is of equal importance for understanding the molecular mechanisms behind the successful hunting strategies of these marine gastropods. Interestingly, many cone snail species contain genes that encode proteins that are highly similar to the α-helical pore-forming toxins, actinoporins, from sea anemones. These conoporins have so far been detected in vermivorous and piscivorous snails, where they often represent a significant portion of the venom composition, both at the transcript and at the protein level. While this suggests that conoporins have an important biological role in the cone snail venom, their exact function remains unknown. Thus far, Cand from *T. andremenezi* is the only conoporin to be experimentally characterized, showing features distinct from typical actinoporins, such as non-specific binding to lipid sphingomyelin and formation of hexameric pores. In order to test the several hypotheses regarding the exact function of these pore-forming proteins in cone snails, additional conoporins need to be experimentally characterized, comparing the properties of conoporins from different species as well as different conoporin isoforms from the same species. Moreover, studies focusing on cone snail venom expression can shed light on other venom components that might synergistically act with conoporins, enhancing the envenomation process.

## Figures and Tables

**Table 1:** List of conoporin sequences discovered in cone snails with different diets.

Diet	Clade	Nr. of Species	Nr. of Sequences	References
Vermivorous	*Virroconus*	3	25	[68,71,78]
	*Lautoconus*	5	10	[79,80]
	*Strategoconus*	2	8	[78,81]
	*Rhizoconus*	1	8	[71]
	*Kalloconus*	2	6	[79,82] ^1^
	*Lividoconus*	2	5	[71,83]
	*Rhombiconus*	1	2	[71]
	*Splinoconus*	2	2	[84]
	*Turriconus*	1	1	[85]
	*Dendroconus*	1	1	[86]
Piscivorous	*Pionoconus*	3	12	[67,87,88,89]
	*Gastridium*	1	9	[90]
	*Chelyconus*	1	6	[91]
Molluscivorous	/	0	0	/

^1^ (Herráez-Pérez, A.; Tenorio, M.J.; Galindo, J.C.; Zardoya, R., personal communication, 5 April 2024).

**Table 2:** Proposed hypotheses for the biological roles of conoporins.

Biological Role	Description	References
Disruption of cells and epithelia	Pore formation causes cell swelling and lysis due to colloid-osmotic shock. This leads to disintegration of epithelia, allowing for smaller conotoxins to pass through epithelial barriers (e.g., blood-brain barrier).	[92,93,94,95,96,97,98,99]
Entry of small conotoxins into cells through pores	Pores formed by conoporins allow for the translocation of smaller conotoxins into the target cell.	[100,101,102,103,104,105,106]
Part of the immune system	Conoporins are employed in the destruction of pathogens by forming pores on their cell membranes, causing lysis.	[27,62,73,75,76,80,107,108,109,110,111,112,113,114,115,116,117]
Digestion	Conoporins facilitate the breakdown of digested tissue by lysing digested cells via pore formation.	[40,118,119]

## Data Availability

No new data were created or analyzed in this study.
